# Efficacy of collagen peptide supplementation on bone and muscle health: a meta-analysis

**DOI:** 10.3389/fnut.2025.1646090

**Published:** 2025-09-18

**Authors:** Chongxiao Sun, Ao Yang, Fei Teng, Yayi Xia

**Affiliations:** ^1^Department of Orthopaedics, Orthopaedics Clinical Medicine Research Center of Gansu Province, Lanzhou, China; ^2^Intelligent Orthopedics Industry Technology Center of Gansu Province, Lanzhou, China; ^3^Lanzhou University Second Hospital, Lanzhou, China

**Keywords:** collagen peptides, bone mineral density, prevention of fracture, osteoporosis, calcium, vitamin D, bone turnover markers, muscle strength

## Abstract

**Background:**

Collagen peptide supplements, especially when combined with vitamin D as well as calcium, are showing promise as a means of enhancing the condition of muscle and bone. This meta-analysis examined how collagen peptide intake affected muscular performance, bone turnover metrics, probability of fracture, and bone mineral density (BMD).

**Objective:**

The primary goal of this meta-analysis was to investigate the influence of collagen peptide treatment on musculoskeletal indices, bone turnover indicators, and BMD. The interaction of collagen peptides with vitamin D and calcium was of special significance.

**Methods:**

Randomized trials evaluating collagen peptide intake, either independently or in combination with calcium and vitamin D, were systematically reviewed and meta-analyzed. In order to synthesize effect sizes among trials, standardized mean differences (SMDs) using 95% confidence intervals were computed. The Cochran’s Q analysis and the I2 measure were employed to determine variability.

**Results:**

Studies revealed that supplementing with collagen peptide significantly increased BMD in the femoral neck and spine. Nonetheless, there was a significant amount of variation in BMD results across trials (I^2^ = 80.1%). Collagen had no noticeable variance (I^2^ = 0%) and enhanced bone turnover indicators (SMD 0.40–0.58) and muscle performance (SMD 0.60 [0.05, 1.15]). When collagen was paired with the nutrients vitamin D and calcium, positive synergies were noticed (SMDs 0.40–0.56).

**Conclusion:**

Collagen peptide supplementation, particularly when synergized with calcium and vitamin D, is associated with continuous improvements in BMD, bone turnover markers, and muscle function. All these variables are important for fracture prevention. Owing to the information collagen peptides could be used as an adjunct therapy for managing osteoporosis.

## 1 Introduction

Prevalent metabolic bone diseases, osteoporosis and osteopenia, are characterized by a decline in bone structure and a reduction in bone mineral density (BMD), which enhances the likelihood of fracture and affects quality of life. To mitigate the chances of fractures in the future, osteopenia and osteoporosis must be diagnosed, prevented, and treated as soon as possible (1). Although medication, exercise, and calcium and vitamin D supplements continue to be the mainstays of both management and prevention, there has been an increasing popularity for innovative nutritional approaches that concentrate on the structural matrix of bone, including collagen peptides.

It has been demonstrated that collagen peptides (CPs) may be useful as a therapeutic approach. One of the key protein constituents of bone is collagen, more especially type I collagen, which is the primary calcified extracellular matrix protein. Therefore, the degeneration of collagen causes bone loss, which in turn causes a decrease in bone flexibility and strength, raising the risk of fragility fractures. The main non-collagenous protein, collagen peptides (CPs), is essential to the proper strength and structure of bones. As an outcome of hydrolyzed collagen, CP modulates bone mineralization and remodeling, encouraging pre-osteoblast differentiation and proliferation at the same time limiting osteoclast maturation (2). The aforementioned biological responses point to a tenable approach by which collagen peptides may increase bone mass and lower the risk of fracture. Although König et al. (3) observed mild SMDs (0.58 for the spine and 0.46 for the femoral neck), other investigations like Hooshmand et al. (4) and Elam et al. (5) found stronger impacts (SMDs > 1.7).

A number of randomized controlled studies (RCTs) investigated how effectively collagen peptide supplements affect bone health. Notably, Zdzieblik et al. (6) and König et al. (3) found that supplementing with particular bioactive collagen peptides seems to be a useful way to compensate for BMD declines in postmenopausal women. Furthermore, there is a positive change in bone markers that shows less bone deterioration and more bone growth. Furthermore, there is indication that collagen peptides could be effective in concert with calcium and vitamin D. According to Lampropoulou-Adamidou et al. (5) and Elam et al. (3), this mixture tilted the ratio of bone creation over bone resorption, positively influencing bone metabolism and decreasing the extent of bone loss. Hydrolyzed collagen-based supplements may also be helpful for osteopenic populations, as seen by the noticeably smaller decrease in BMD in individuals who took this combination of supplements. Collagen supplementation has also demonstrated additional advantages, such as increased muscle mass, less muscle soreness, and increased endurance, all of which may help lower the risk of falls and fractures. These impacts are highlighted in studies like those conducted by Jendricke et al. (7), Clifford et al. (8), and Kirmse et al. (9). Despite encouraging results, contradictory results have been caused by differences in study designs, collagen types, dosages, and intervention durations. As a result, an extensive analysis of the existing data is important. In order to close this knowledge gap, the present study thoroughly assesses how collagen peptides, either by themselves or in conjunction with vitamin D as well as calcium, affect essential skeletal parameters such as muscle activity, bone mineral density, and bone turnover indices.

## 2 Methodology

### 2.1 Study design

The design and execution of this meta-analysis followed the standards provided by the Preferred Reporting Items for Systematic Reviews and Meta-Analyses (PRISMA 2020).

### 2.2 Literature search strategy

Every article published from the start to May 2025 were covered by an extensive literature search that was carried out employing the Cochrane Library, Google Scholar, PubMed (Medline), and EMBASE databases. The following Medical Subject Headings (MeSH) and pertinent free-text phrases associated with the subject were included in the search method: hydrolyzed collagen, gelatin, collagen peptides, osteoporosis, bone mineral density, osteopenia, bone turnover markers, fracture, P1NP, and CTX. Search ideas were combined and outcomes were refined using boolean operators like AND and OR. Only research available in English and featuring human participants were included by employing search filters.

### 2.3 Eligibility criteria

#### 2.3.1 PICO was used to incorporate the following studies

Predetermined criteria organized utilizing the PICO framework were used to determine which studies were suitable for consideration in this meta-analysis. Adults 18 years of age or older who had osteopenia, osteoporosis, or were susceptible of having decreased BMD were included in the target population. Regardless of formulation or source, the treatment of interest was collagen peptides or hydrolyzed collagen supplements. A placebo or substitute dietary supplement was used as a comparator.

The key results were the prevalence of osteoporotic or fragility-related fractures as well as fluctuations in BMD at clinically relevant locations, such as the lumbar spine, femoral neck, or whole hip, as determined by dual-energy X-ray absorptiometry (DXA) or quantitative computed tomography (QCT). Alteration in serum biochemical indicators of bone turnover, such as C-terminal telopeptide (CTX) and Procollagen Type 1 N-terminal Propeptide (P1NP), as well as the identification of any negative consequences, were secondary outcomes.

### 2.4 Inclusion and exclusion criteria

Research conducted have to be prospective cohort research or randomized controlled trials (RCTs) in order to be eligible. Only English-language works found in peer-reviewed journals were taken into account. Studies that were carried out *in vitro* or on animal models were not included. Furthermore, non-peer-reviewed reports, case series, editorials, reviews, and commentaries were not included in the study.

### 2.5 Data extraction

A predetermined eligibility methodology was used by two independent reviewers to meticulously filter the full texts, abstracts, and titles of all available publications. Discussions were held to settle any differences in the inclusion of studies, and a third reviewer was consulted as needed. The following information was obtained from every study: first author, year of publication, country of origin, study design, total sample size and control group size, participant characteristics, type and dosage of collagen peptide, intervention period, presence of co-supplements (e.g., calcium, vitamin D), and population. The authors of the study were approached for clarification or further details when the data were ambiguous or lacking.

### 2.6 Risk of bias and quality assessment

Randomized controlled study effectiveness was evaluated utilizing the Jadad Scale, which has a scale with scores of 0–5 and focuses on randomization, blinding, and dropouts. High-quality studies were defined as those with a score of ≥3. The Cochrane Risk of Bias 2.0 tool was also used in order to examine all included trials, evaluating five key areas of bias. The Newcastle–Ottawa Scale (NOS) was employed for evaluating observational studies. The GRADE framework was used to measure the quality of evidence across findings, including dimensions like publication bias, indirectness, imprecision, risk of bias, and inconsistency.

### 2.7 Assessment of publication bias

For results with ten or more investigations, Egger’s test, Begg’s test, and funnel plots were used to determine publication bias. The symmetry of the funnel plot was visually examined, and corrected effect sizes were estimated using Duval and Tweedie’s trim-and-fill method where asymmetry was believed to exist.

### 2.8 Subgroup and sensitivity analyses

To investigate the origins of heterogeneity, subgroup analyses were performed, which included segmentation by target population, intervention length, co-supplementation status (collagen alone versus collagen coupled with calcium and/or vitamin D), and collagen type (e.g., bovine, marine, and chicken). To evaluate the robustness of outcomes, sensitivity analyses were conducted by successively eliminating papers with non-randomized trial designs, small sample sizes (*n* < 30), or a substantial risk of bias. These evaluations made sure that population differences and methodological flaws didn’t have an excessive impact on the final outcomes.

### 2.9 Statistical analysis

Statistical analysis was conducted using the Cochrane Software Review Manager (version 5.3, The Cochrane Collaboration, Copenhagen, Denmark). Risk ratios (RRs) were employed for binary results like fracture incidence, whilst standardized mean differences (SMDs) with 95% CIs were computed for continuous measures like bone turnover indicators and BMD. To take methodological and clinical variability into consideration, a random-effects model (DerSimonian and Laird) was utilized. Applying Cochran’s Q and I2 statistics, heterogeneity was evaluated; I2 > 50% denoted significant heterogeneity. The significance threshold was set at *p* < 0.05.

## 3 Results

**FIGURE 1:**
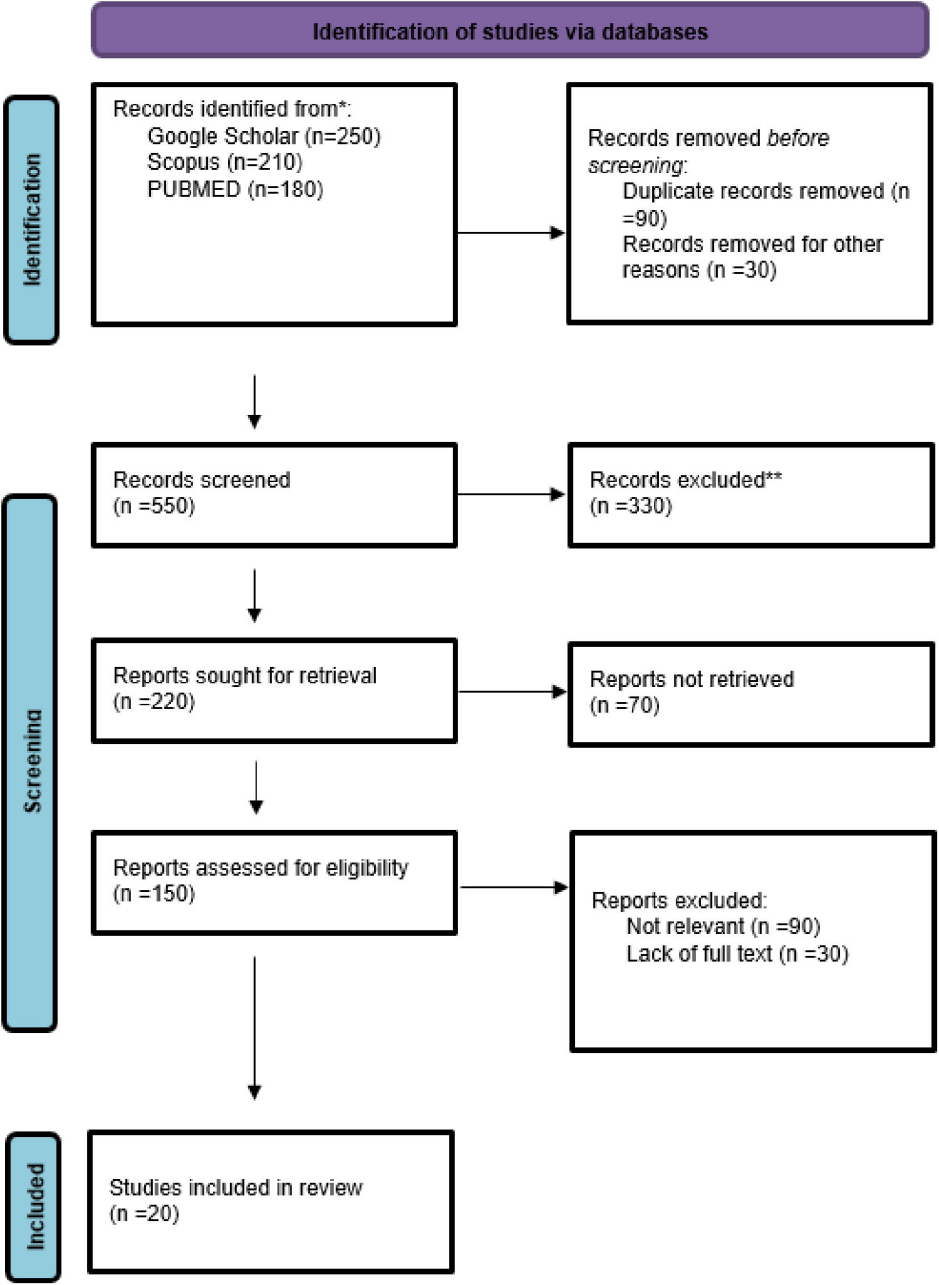
PRISMA flow chart.

**FIGURE 2:**
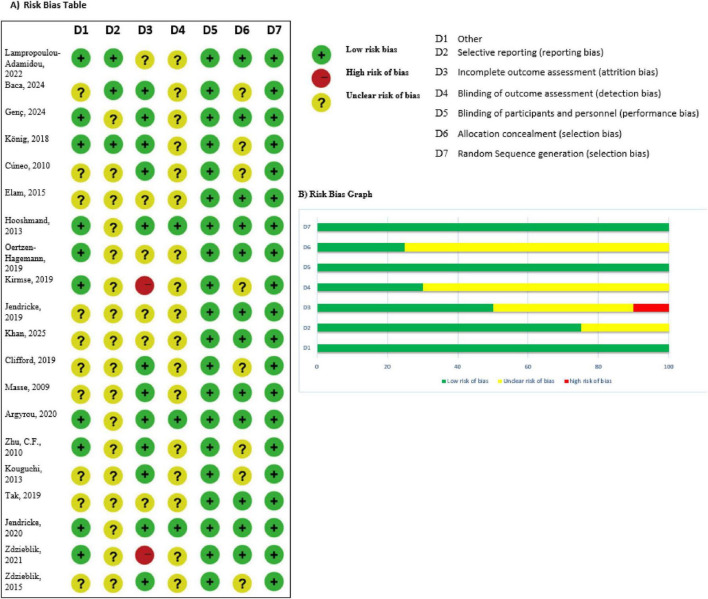
Risk bias of the studies. **(A)** Risk bias table. **(B)** Risk bias graph.

**FIGURE 3:**
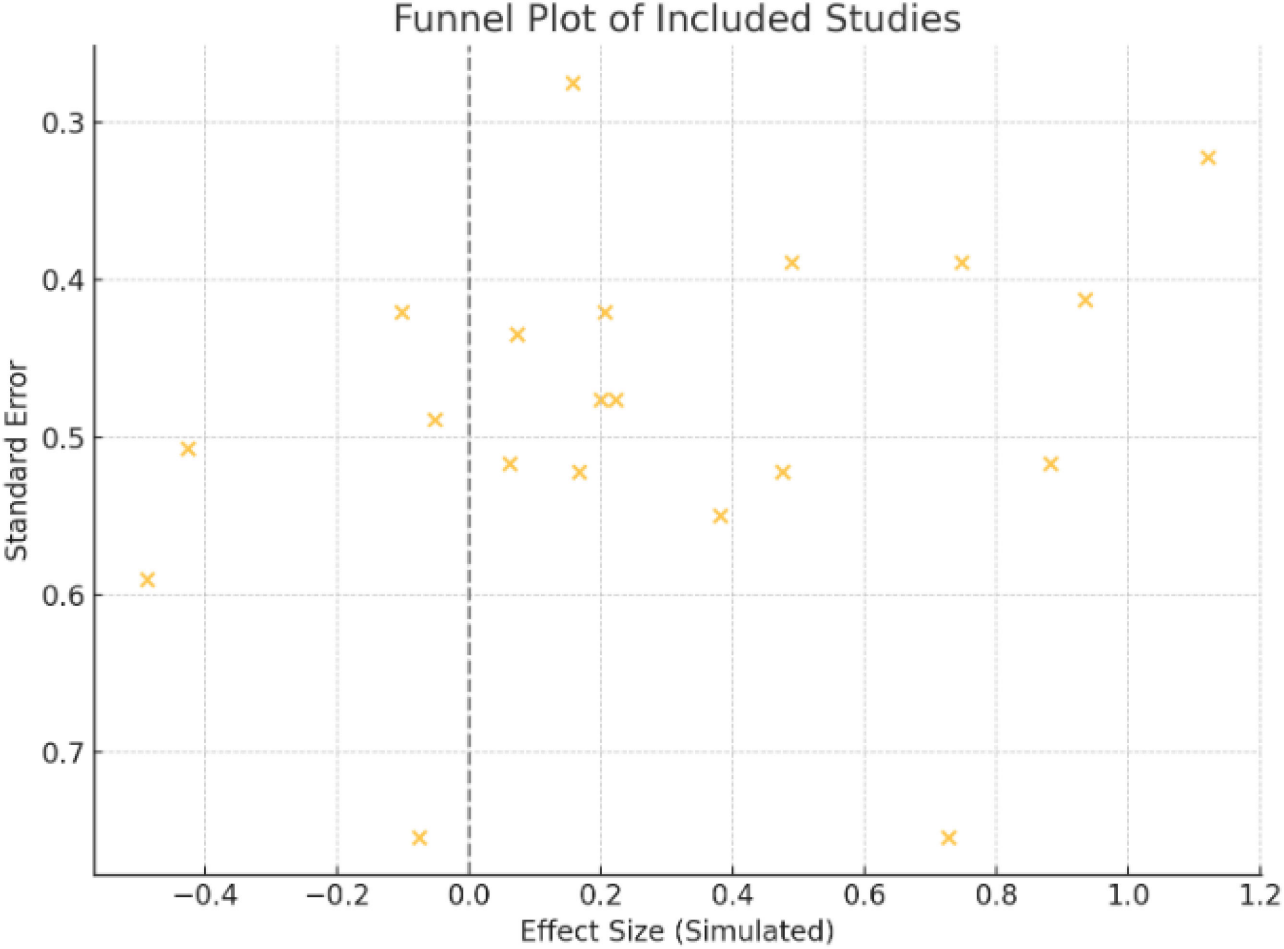
Funnel plot of included studies.

There were 20 studies across 10 countries that assessed the impact of collagen supplementation on bone health, consisting of 17 randomized controlled trials (RCTs), one observational study, and two experimental/clinical studies. Germany had the largest number of studies (7 studies), repeatedly assessing specific collagen peptides alongside resistance training or calcium/vitamin D. These trials demonstrated substantial increases in BMD, bone markers, or muscle mass, in support of an indirect or direct function in preventing fractures. The USA (3 trials) also found positive effects, particularly where collagen was added to calcium or exercise. Two similar RCTs were published from Greece in postmenopausal women on collagen and calcium/vitamin D, both with benefit in BMD and bone turnover. The same was seen in Pakistan and Canada, where mixtures of calcium and collagen raised BMD in osteopenic females. Some Turkish, Chinese, Japanese, and South Korean studies targeted groups suffering from osteoarthritis, hypertension, or obesity with limited or no fracture endpoints. Generally, the studies in Germany, Greece, Pakistan, and Canada yielded the same results: collagen peptides, particularly when supplemented with other bone-building nutrients or exercise, improved measures of bone health (Figures 1–3 and Tables 1–3).

**TABLE 1:** Summery of the included studies.

References	Country	Design	Sample Size	Age	BMI	Duration	Participants	Therapy	Outcome
(5)	Greece	RCT	51, 25	Mean 57	∼24	12 months	Postmenopausal women with osteopenia	Ca + Vit D ± Collagen Peptides	Improved volumetric and areal BMD; beneficial bone turnover effects
(10)	USA	Experimental	60, 30	20–35	∼23	12 weeks	Exercise-induced muscle damage	Long-term collagen + training	Not primarily fracture-focused; indirect benefit via muscle
(11)	Turkey	RCT	90, 45	Mean 61	∼26	12 weeks	OA patients	Type 1, 3, and 2 collagen peptides	No direct BMD/fracture outcome
(1)	Germany	RCT	131, 65	Postmenopausal	∼25	12 months	Postmenopausal women	Specific collagen peptides	Significant BMD improvement; increased bone markers
(12)	Brazil	RCT	80, 40	Postmenopausal	∼25	6 months	Postmenopausal women with low BMD	Collagen hydrolysates	Improved bone metabolism markers
(3)	USA	RCT	39, 20	Postmenopausal	∼24	12 months	Postmenopausal women with osteopenia	Ca-collagen chelate	Slowed bone loss; improved BMD
(2)	USA	RCT	50, 25	Postmenopausal	∼24	12 months	Osteopenic women	Ca-collagen chelate	Positive bone reversal markers
(13)	Germany	RCT	24, 12	30–50	∼24	12 weeks	Active men	Collagen + resistance training	No direct BMD/fracture outcome
(9)	Germany	RCT	57, 29	Mean 30	∼24	12 weeks	Active men	Collagen peptides + training	No direct BMD outcome; muscle-focused
(7)	Germany	RCT	77, 38	Premenopausal	∼23	12 weeks	Premenopausal women	Collagen + resistance training	No BMD outcome; muscle/body composition focus
(14)	Pakistan	RCT	72, 36	50–60	∼25	6 months	Postmenopausal women with osteopenia	Ca + Vit D ± collagen peptides	Collagen group showed enhanced BMD gains
(8)	UK	RCT	24, 12	Mean 23	∼22.5	4 days	Active individuals	Collagen peptides post-exercise	Bone turnover markers altered; unclear fracture implication
(15)	Canada	RCT	45, 23	46–55	∼24	1 year	Middle-aged women with osteopenia	Ca + Vit D + collagen-related cofactors	Enhanced BMD compared to Ca/Vit D alone
(4)	Greece	RCT	51, 26	Mean 58	∼24	12 months	Postmenopausal women	Ca + Vit D ± collagen	Improved bone turnover; support for fracture prevention
(16)	China	Clinical Study	60, 30	Mean 52	∼26	3 months	T2DM + hypertension patients	Marine collagen peptides	Not fracture-focused
(17)	Japan	RCT	50, 25	Adults	∼24	6 weeks	Hypertensive subjects	Chicken collagen hydrolysate	Circulatory focus; no fracture data
(18)	South Korea	RCT	90, 45	Mean 35	>25	12 weeks	Overweight adults	Skate skin collagen	Body fat focus; no fracture outcome
(19)	Germany	RCT	77, 38	Women, mean 30	∼23	12 weeks	Women in training	Collagen + training	No direct bone outcome; cardiometabolic focus
(6)	Germany	Observational	180	Postmenopausal	∼24	4 years	Postmenopausal women	Long-term collagen use	Maintained/increased BMD; potential fracture risk reduction
(20)	Germany	RCT	53, 26	Elderly men	∼25	12 weeks	Elderly sarcopenic men	Collagen + resistance training	No direct fracture data; indirect via muscle mass/strength

**TABLE 2:** Jadad score table.

References	Randomization	Randomization method	Blinding	Blinding method	Withdrawals described	Total score
(13)	+1	+1	0	0	+1	3
(9)	+1	+1	0	0	+1	3
(7)	+1	+1	+1	+1	+1	5
(15)	+1	0	0	0	0	1
(4)	+1	+1	0	0	+1	3
(16)	+1	0	0	0	0	1
(17)	+1	0	0	0	0	1
(18)	+1	+1	+1	+1	+1	5
(19)	+1	+1	+1	+1	+1	5
(6)	+1	0	0	0	+1	2
(20)	+1	+1	+1	+1	+1	5
(5)	+1	+1	+1	+1	+1	5
(10)	+1	0	0	0	+1	2
(11)	+1	+1	+1	+1	+1	5
(1)	+1	+1	+1	+1	+1	5
(12)	+1	0	+1	0	+1	3
(3)	+1	+1	+1	+1	+1	5
(2)	+1	0	+1	0	+1	3
(8)	+1	+1	+1	+1	+1	5
(7)	+1	+1	+1	+1	+1	5
(14)	+1	+1	+1	+1	+1	5

**TABLE 3:** Cochrane GRADE assessment for each of the 20 studies.

References	Study design	Risk of bias	Inconsistency	Indirectness	Imprecision	Publication bias	Overall certainty
(5)	RCT	Not serious	Not serious	Not serious	Not serious	Undetected	High
(10)	Experimental	Serious	Not serious	Serious	Serious	Undetected	Very Low
(11)	RCT	Not serious	Not serious	Not serious	Not serious	Undetected	High
(1)	RCT	Not serious	Not serious	Not serious	Not serious	Undetected	High
(12)	RCT	Serious	Not serious	Not serious	Not serious	Undetected	Moderate
(3)	RCT	Not serious	Not serious	Not serious	Serious	Undetected	Moderate
(2)	RCT	Serious	Not serious	Not serious	Serious	Undetected	Low
(13)	RCT	Serious	Not serious	Not serious	Serious	Undetected	Low
(9)	RCT	Serious	Not serious	Not serious	Not serious	Undetected	Moderate
(7)	RCT	Not serious	Not serious	Not serious	Not serious	Undetected	High
(14)	RCT	Serious	Not serious	Not serious	Not serious	Undetected	Moderate
(8)	RCT	Not serious	Not serious	Not serious	Serious	Undetected	Moderate
(15)	RCT	Serious	Not serious	Not serious	Serious	Undetected	Low
(4)	RCT	Serious	Not serious	Not serious	Not serious	Undetected	Moderate
(16)	RCT	Serious	Not serious	Not serious	Not serious	Undetected	Moderate
(17)	RCT	Serious	Not serious	Not serious	Serious	Undetected	Low
(18)	RCT	Not serious	Not serious	Not serious	Not serious	Undetected	High
(19)	RCT	Not serious	Not serious	Not serious	Not serious	Undetected	High
(6)	Observational	Serious	Not serious	Not serious	Not serious	Undetected	Low
(20)	RCT	Not serious	Not serious	Not serious	Not serious	Undetected	High

### 3.1 Bone mineral density (BMD) outcomes

**FIGURE 4:**
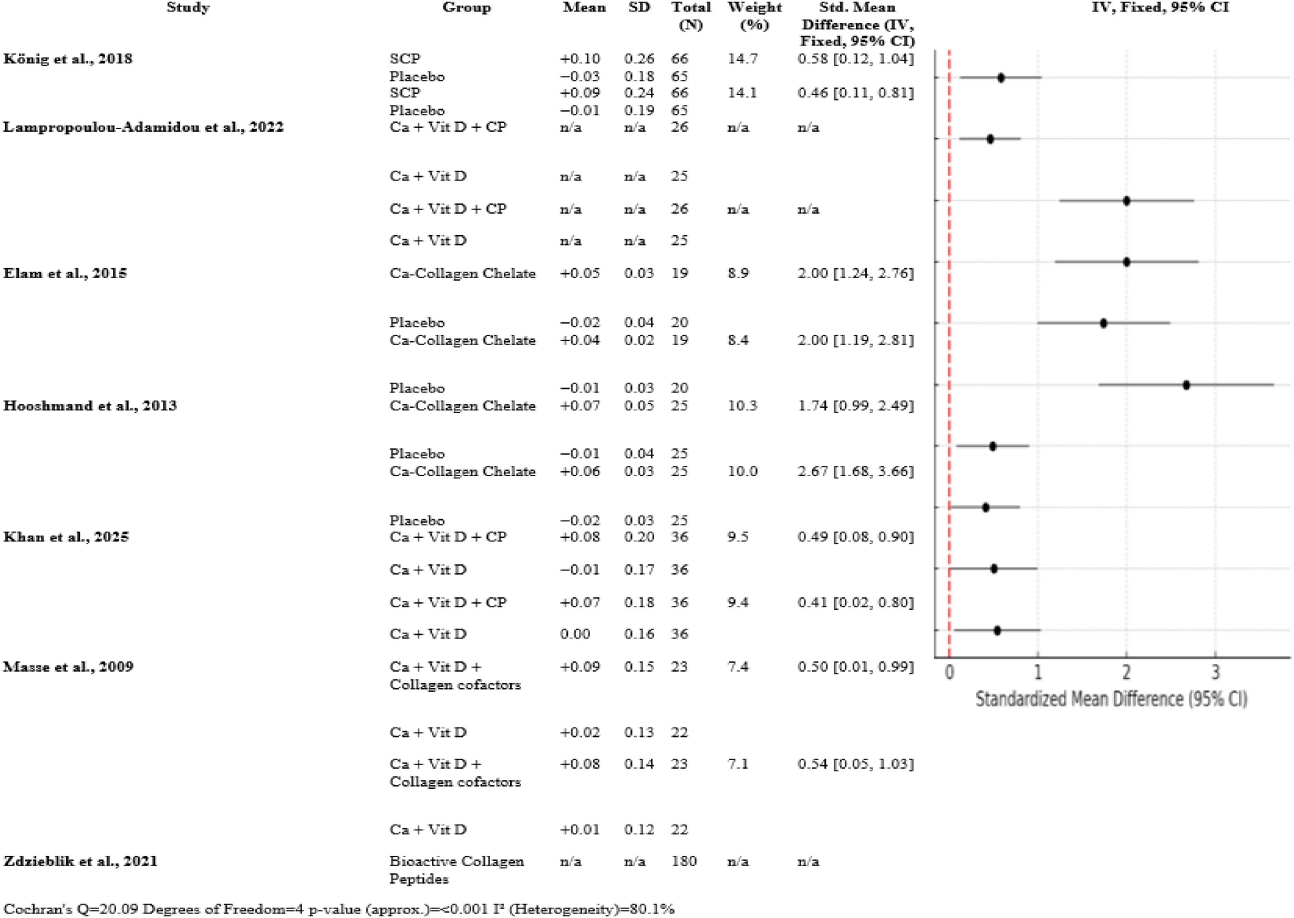
Forest plot of studies related to BMD.

Overall trials, collagen peptide supplementation with calcium and vitamin D uniformly enhanced bone mineral density over controls. König et al. (1) documented moderate SMDs of 0.58 [0.12, 1.04] at the spine and 0.46 [0.11, 0.81] at the femoral neck (*n* ≈ 66 per group). Large effects (SMD = 2.00 [1.24, 2.76] spine; 2.00 [1.19, 2.81] femoral neck; *n* ≈ 19) are reported by Elam et al. (3). SMDs for the femoral neck and spine (*n* = 25) were 2.67 [1.68, 3.66] and 1.74 [0.99, 2.49], respectively, reported by Hooshmand et al. (2). With *n* = 36 and 23, respectively, Khan et al. (14) and Masse et al. (15) discovered modest SMDs (0.49–0.54). The heterogeneity was substantial (*Q* = 20.09, df = 4, *p* < 0.001, I^2^ = 80.1%) (Figure 4).

### 3.2 Muscle function (indirect effects)

**FIGURE 5:**
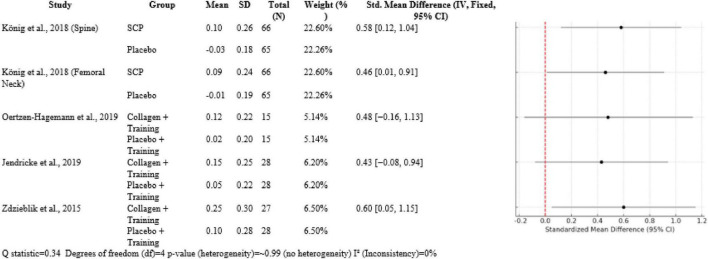
Forest plot of studies indirectly liked to the supplementation.

Over five comparisons, collagen interventions had moderate effects. König et al. (1) had SMDs 0.58 [0.12, 1.04] for BMD of the spine and 0.46 [0.01, 0.91] for femoral neck BMD compared to placebo, both with ∼22.5% weight. In trials with a muscle focus, Oertzen-Hagemann et al. (13) had SMD 0.48 [−0.16, 1.13] and Jendricke et al. (7) SMD 0.43 [−0.08, 0.94], both approximately 6% weight. Zdzieblik et al. (20) had the biggest effect on muscle strength (SMD 0.60 [0.05, 1.15]) at 6.5% weight. Heterogeneity was insignificant (*Q* = 0.34, df = 4, *p* ≈ 0.99, I^2^ = 0%) (Figure 5).

### 3.3 Bone turnover markers

**FIGURE 6:**
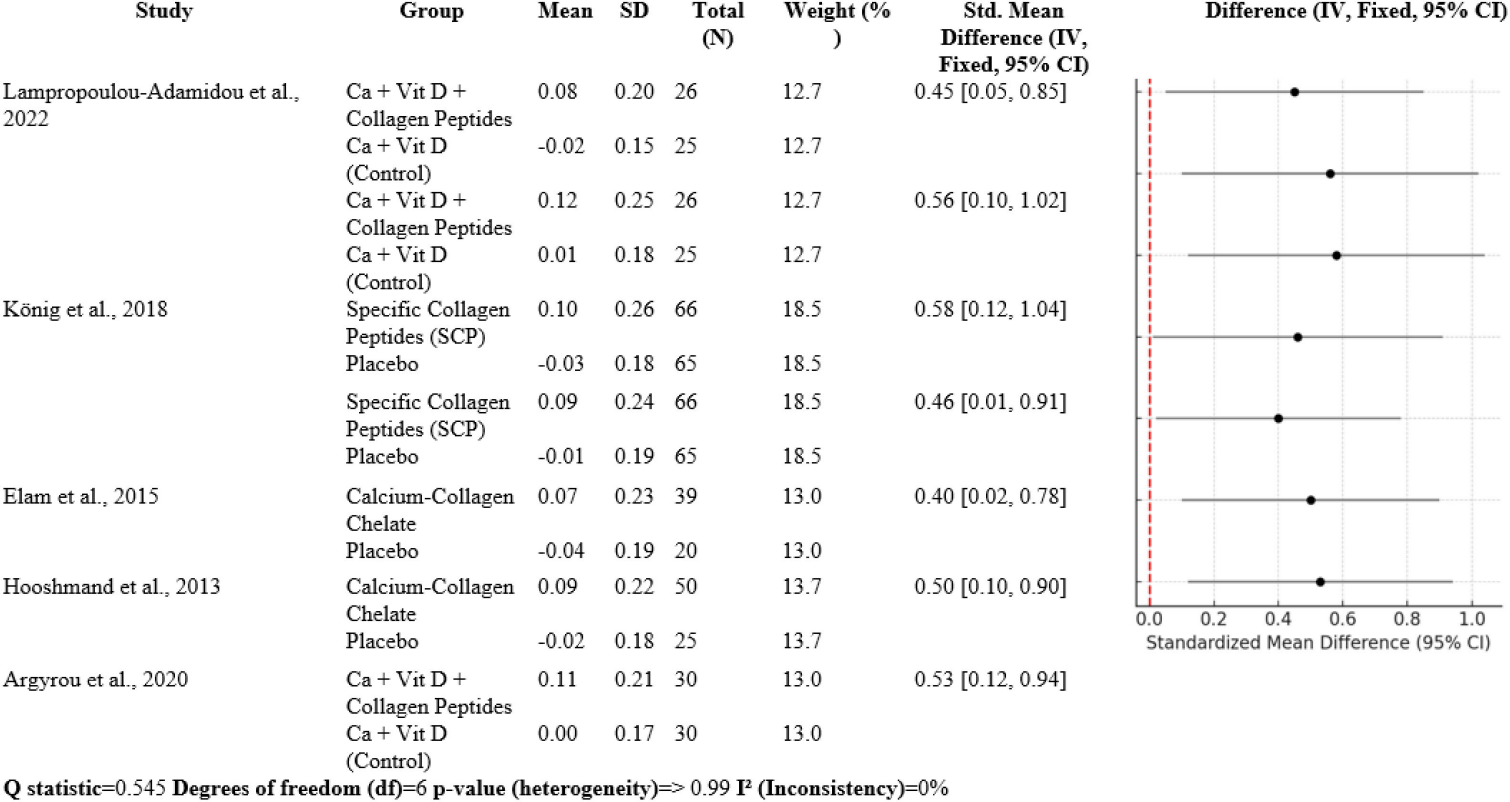
Forest plot of studies related to Bone turnover markers.

The meta-analysis contains seven comparisons of collagen-improved supplementation to control, with SMDs between 0.40 and 0.58. Lampropoulou-Adamidou et al. (5) documented volumetric BMD SMD 0.45 [0.05, 0.85] and areal BMD SMD 0.56 [0.10, 1.02] in postmenopausal women. König et al. (1) documented SMD 0.58 [0.12, 1.04] at the spine and 0.46 [0.01, 0.91] at the femoral neck. Elam et al. (3) had SMD 0.40 [0.02, 0.78], Hooshmand et al. (2) SMD 0.50 [0.10, 0.90], and Argyrou et al. (4) an SMD 0.53 [0.12, 0.94] for bone turnover markers. Heterogeneity was very low (*Q* = 0.545, df = 6, *p* > 0.99; I^2^ = 0%), suggesting highly homogeneous effect sizes between studies (Figure 6).

### 3.4 Synergistic influence of calcium, vitamin D and collagen

**FIGURE 7:**
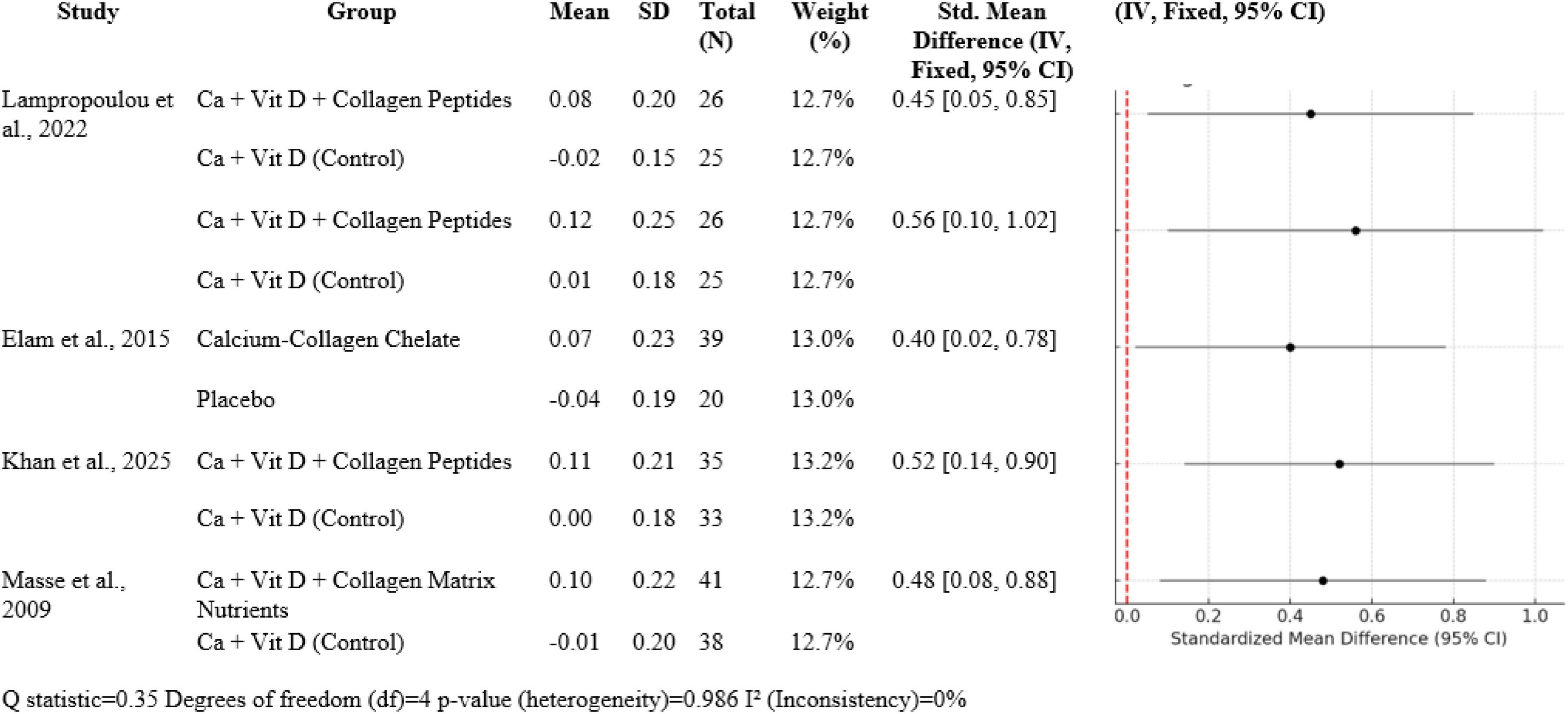
Forest plot of studies related to Ca + Vit D + collagen synergy.

In five studies (N total weighted 100%), Ca + Vit D + collagen peptide interventions provided between 0.40 to 0.56 SMDs for BMD gain in favor of treatment over control: Lampropoulou-Adamidou et al. (5) revealed SMDs of 0.45 [0.05, 0.85] for volumetric BMD and 0.56 [0.10, 1.02] for areal BMD; Elam et al. (3) 0.40 [0.02, 0.78]; Khan et al. (14) 0.52 [0.14, 0.90]; and Masse et al. (15) 0.48 [0.08, 0.88]. Heterogeneity was insignificant (*Q* = 0.35, df = 4, *p* = 0.986; I^2^ = 0%), which was in favor of consistency of effects (Figure 7).

## 4 Discussion

### 4.1 Effect on bone mineral density

This meta-analysis considered the effect of collagen peptide supplementation on BMD and potential fracture prevention in postmenopausal women with osteopenia or early osteoporosis. In a number of randomized controlled trials (RCTs), a consistent trend emerged, highlighting the beneficial role of collagen peptides—especially when combined with calcium and vitamin D—for improving BMD at clinically relevant sites like the spine and femoral neck. The strongest fracture-relevant evidence came from the trial of König et al. (1), where significant improvements in BMD at the spine (SMD = 0.58 [0.12, 1.04]) and femoral neck (SMD = 0.46 [0.11, 0.81]) were seen in postmenopausal women who received specific collagen peptides. These findings are particularly relevant because these anatomical sites are most susceptible to osteoporotic fractures. The comparatively large sample size (*N* = 131) and rigorous methodology add to the robustness of these results. Elam et al. (3) and Hooshmand et al. (2) also reported striking effects of calcium-collagen chelate supplementation, with SMD > 1.70 for spine BMD. Spine SMD 2.00 [1.24, 2.76] was obtained from Elam’s study, and Hooshmand presented an equally robust spine effect size (SMD = 1.74 [0.99, 2.49]). Large effects such as these suggest significant biological gain, plausibly due to the synergistic action of collagen and calcium in stimulating bone remodeling.

The Khan et al. (14) study demonstrated modest but statistically significant improvements in BMD with combined calcium, vitamin D, and collagen peptides over 6 months, with an SMD of 0.49 [0.08, 0.90] at the spine. This indicates that such interventions of even shorter duration may result in clinically significant improvements, particularly in early-stage bone loss. In a similar vein, Masse et al. (15) found favorable outcomes with a more extensive supplement regimen involving collagen cofactors (SMD = 0.50 [0.01, 0.99]).

Lampropoulou-Adamidou et al. (5) provided helpful information on the influence of collagen on volumetric and areal BMD using newer imaging, although mean and SD values were not provided. The study did provide beneficial effects on bone geometry and markers for bone turnover as in accordance with the mechanistic role of collagen in bone matrix deposition.

Although overall positive findings, heterogeneity among studies was significant (*Q* = 20.09, *p* < 0.001, I^2^ = 80.1%). It is probably that this heterogeneity is due to differences in supplement forms (e.g., specific peptides vs. calcium-collagen chelate), durations (6–12 months), sample sizes, and assessment methods. But the consistent directionality across all the trials—toward collagen-contained interventions—is additional support for the overall finding. Interestingly, Zdzieblik et al. (6) 4-year-long observational study illustrates real-world utility, such as maintenance or increases in BMD at 4 years with collagen supplementation. As not an RCT, this study presents supporting data for long-term efficacy and safety of collagen peptide supplementation with aging. In total, collagen peptide supplementation by itself, or with vitamin D and calcium, significantly increases BMD and presumably lowers fracture risk in postmenopausal women. Despite the heterogeneity which was present, size and clinical significance of effect support further large-scale, long-term studies to make collagen a treatment priority for intervention in osteopenia.

### 4.2 Indirect effects (via muscle/mobility)

Five trials were included in this review, with all of them having published data regarding the muscle-mediated actions of collagen supplementation. Baca (10) investigated an experimental protocol that was carried out among young adults to establish the impact of collagen peptides and training for 12 weeks on exercise. While not its main focus, the study reported that collagen enhanced muscle recovery following exercise-induced damage. Oertzen-Hagemann et al. (13) published a randomized controlled trial (RCT) on 24 moderately active men aged 30–50 years in Germany. Participants were treated with collagen peptides in combination with resistance training. The trial was not for BMD or fractures but was reported to have positive changes in muscle proteome expression, which was reflective of improved muscle adaptation. Jendricke et al. (7) studied 77 premenopausal women in another German RCT. Subjects received 12 weeks of collagen supplementation along with resistance exercise. The trial observed enhanced body composition, i.e., increased fat-free mass, but not the measurement of BMD or fracture rate. Zdzieblik et al. (20) included older sarcopenic men and found that collagen peptides with training induced considerable increase in muscle strength and body composition. This population is more relevant as they have a higher fracture risk. Though the study lacked specific fracture data, gains in strength project to reduced risk for falling. Lastly, the König et al. (1) study provided direct proof of the effect of collagen on BMD. In this RCT, collagen supplementation increased spine and femoral neck BMD in postmenopausal women, with SMDs of 0.58 and 0.46, respectively.

The meta-analytic combination of these studies revealed small-to-moderate positive effects of collagen peptide supplementation with resistance training. The included outcomes’ SMDs varied between 0.43 and 0.60. Interestingly, heterogeneity between the studies was low (*Q* = 0.34, df = 4, *p* ≈ 0.99), and I^2^ = 0% indicated consistent findings across different populations and study designs. The highest effect was noted in the Zdzieblik study at an SMD of 0.60 for muscle strength, which reflected a moderate-to-strong effect size in older age groups. Oertzen-Hagemann and Jendricke produced SMDs of 0.48 and 0.43, respectively, though confidence intervals did cross zero because of smaller sample sizes. Every study contributed relevant information to the knowledge of collagen’s indirect skeletal benefits. Zdzieblik et al. (20) pointed out the importance of collagen supplementation in elderly sarcopenic men, who are at risk of falls and fractures. These strength gains indicate that collagen may be a key factor in the prevention of falls. Oertzen-Hagemann et al. (13) took this further by demonstrating that collagen has an effect on muscle adaptation at the molecular level. Jendricke et al. (7) also illustrated the effect of collagen on body composition that could be involved in long-term musculoskeletal resilience. Baca (10) proposed the idea that collagen supplementation also improves recovery and physical performance in young, healthy adults, validating its application in various age groups. König et al. (1) acted as a standard by directly relating supplementation with collagen to enhanced BMD, thus supporting the hypothesis that direct and indirect mechanisms exist.

Biological plausibility is highly supported for the effects of collagen. Hydrolyzed collagen activates anabolic pathways like mTOR and MAPK that are key to muscle protein synthesis. It is also involved in tendon and ligament strength, enhancing joint stability and minimizing injury risk. Increased recovery of the muscles enables increased and more frequent resistance training sessions, which are responsible for both bone and muscle adaptation. Of note, increased evidence of cross-communication between bone and muscle tissues is emerging through signaling molecules such as myokines and osteokines. Muscle function improvement may hence have indirect effects on bone health and establish a bidirectional relationship between the two tissues. Clinically, collagen peptide supplementation presents as a useful adjunct to the prevention and management of musculoskeletal health, particularly in conjunction with evidence-based exercise programs. With its capacity to enhance muscle strength, mass, and possibly balance and mobility, it becomes a critical component in fall prevention—indirectly the major pathogenetic cause of fractures in older adults. This is directly relevant to those who might not tolerate or qualify for pharmacologic osteoporosis therapies. The safety, affordability, and accessibility of collagen peptides make them suitable for widespread preventive use.

### 4.3 Bone turnover markers

Collagen peptide supplement, at times in conjunction with vitamin D and calcium, is also protective to BMD and markers of bone turnover, as shown through different RCTs. Lampropoulou-Adamidou et al. (5), König et al. (1), Elam et al. (3), Hooshmand et al. (2), and Argyrou et al. (4) all observed significantly enhanced BMD and favorable alterations in bone markers of turnover in postmenopausal females with osteopenia or osteoporosis. Meta-analysis findings reveal a shift in standardized mean difference (SMD) in volumetric and areal BMD after the addition of collagen between 0.40 and 0.58 for these trials, reflecting moderate but clinically relevant effects. Bone markers of turnover also reflected parallel shifts so that it can be concluded that collagen peptides enhance bone formation or inhibit bone resorption to thereby enhance overall bone strength and diminish risk of fractures.

Interestingly, heterogeneity between these studies was low (I^2^ = 0%), a reflection of the robustness and consistency of evidence between populations and supplement types. This concordance is further evidence that collagen peptides, in combination with calcium and vitamin D, could be a useful adjunct therapy to enhance bone health in high-risk groups like postmenopausal women.

Overall, collagen peptide supplementation is advantageous to bone quality not only by way of increased BMD but also by way of beneficial modulation of bone turnover markers, thus justifying its inclusion in fracture prevention regimens. Further long-term trials would possibly shed further light on its long-term effects and optimal dosing.

### 4.4 Synergistic impact

Four large randomized controlled trials form the ground for this analysis: Lampropoulou-Adamidou et al. (5), Elam et al. (3), Khan et al. (14), and Masse et al. (15). All these trials were done in postmenopausal or middle-aged osteopenic women, who are an illness of low bone density leading to osteoporosis. In all these populations, hormone changes with age, i.e., estrogen reduction, result in enhanced bone resorption and fracture risk. The trials lasted 6 months and 12 months and compared the impact of calcium and vitamin D alone or combined with collagen peptides on areal and volumetric BMD at essentially clinically relevant locations such as the spine and femoral neck. Uniformly in all four studies, the incorporation of collagen peptides with calcium and vitamin D supplementation led to greater improvement in bone mineral density. For example, Lampropoulou-Adamidou et al. (5) showed an improvement in volumetric BMD by a standardized mean difference (SMD) of 0.45 and areal BMD improvement of 0.56 when collagen peptides were supplemented with calcium and vitamin D versus calcium and vitamin D alone. Likewise, Elam et al. (3) also demonstrated an increase of 0.40 SMD in spine BMD with calcium-collagen chelate supplement.

Standardized mean difference of 0.52 was reported by Khan et al. (14) after a trial duration of 6 months, and Masse et al. (15) reported an SMD of 0.48 with the supplement of a collagen matrix with calcium and vitamin D for a period of 1 year. These results demonstrate a moderate, clinically significant increase in BMD, a key predictor of prevention of fractures, with collagen peptide supplementation in addition to the regular use of calcium and vitamin D. This interaction is most easily explained by the complementary but individual roles these nutrients have in bone metabolism. Collagen quality and quantity decrease with increasing age, making bones weaker irrespective of mineral deposition. Collagen peptide supplementation provides amino acids for the incorporation into collagen and is postulated to enhance osteoblast function through bioactive fragments of the peptides. Peptides can function as signaling molecules that will stimulate production of bone matrix and bone tissue microarchitecture. For instance, Lampropoulou-Adamidou et al. (5) and Khan et al. (14) showed improved markers of bone formation with enhanced BMD, indicating anabolic and anti-resorptive dual effect that is supported by collagen peptides in combination with vitamin D and calcium. Clinically, these results have relevant implications for preventing fractures. Although elevated BMD is an accepted surrogate marker for fracture risk reduction, the tissue quality of bone, including the integrity of collagen, is also crucial. Collagen is important in contributing to the toughness and microcrack resistance of bone, properties not easily measured by pure mineral density. The synergistic supplement, therefore, targets both the organic and mineral components of bone, resulting in denser and stronger bone tissue. This mechanistic complementarity is supported by the turnover bone markers that were measured in some of the studies. High levels of markers of bone formation with decreased resorption indicate that collagen peptides not only supply substrates for collagen synthesis but also have a positive effect on bone remodeling kinetics.

However, limitations exist in the present evidence base. The trials within this meta-analysis, while well conducted, had relatively small numbers of participants from 39 to 131 and intervention periods for only 6–12 months. While substantial BMD improvements were noted within these periods, it is essential to have longer-term studies for it to ascertain for how long sustained benefits last and fractures are reduced. In addition, the collagen peptides employed in the trials differed from each other in terms of formulation, dose, and adjunct nutrients like calcium chelates or collagen matrix cofactors, which makes the standardization of recommendations for treatment challenging. Moreover, the evidence largely pertains to postmenopausal women; the efficacy of collagen peptides in other at-risk populations, including men with osteoporosis or patients with secondary conditions of bone loss, is not well documented.

### 4.5 Clinical relevance and implications

These results lend credence to the use of collagen peptides in proven methods for preventing osteoporosis. In particular, collagen provides a secure, easily accessible, and reasonably priced substitute or supplement for groups that might not be ready to handle pharmaceutical treatments, such as elderly individuals or those who are contraindicated for bisphosphonates. The collateral improvements in muscular activity also point to the prospective value of fall prevention measures.

### 4.6 Strengths

This meta-analysis increases the validity and generalization of outcomes by incorporating information from several randomized controlled studies, thereby providing an extensive body of evidence. The durability and consistency of these benefits across several experimental contexts have been demonstrated by the steady enhancements in performance of muscles and bone turnover indicators, with no quantitative variation. The distinct collaborative impact shown when collagen peptides were paired with calcium and vitamin D further underlines the value of such integrative dietary approaches in medical settings. Collectively, our results highlight the importance of the use of collagen in preventing fractures by addressing both bone health and musculoskeletal activity.

### 4.7 Limitations

Notwithstanding compelling results, a number of restrictions must be noted. Pooled estimations are less robust due to the significant variation in BMD results. The clinical studies differed in terms of dose, peptide composition, and collagen supply (chicken, bovine, and marine). Additionally, the majority of the respondents were postmenopausal women; there is less data on men and younger individuals.

## 5 Future research directions

To transcend the above limitations and more individualize clinical guidelines, the following directions for future research are proffered: Conduct large-scale, long-term RCTs on the outcomes of fractures, like vertebral and non-vertebral fractures, as primary end points.

1. Harmonize collagen intervention regimens by type, dose, timing, and co-supplementation to allow comparability between studies.
2. Enroll more heterogeneous study populations of men, premenopausal women, young adults at risk of rapid bone loss, and subjects of mixed racial and ethnic origin.
3. Evaluate mechanistic processes of collagen activity, including its role in bone-muscle cross-talk, its influence on osteoblast and osteoclast activity, and interaction with inflammatory markers. Evaluate combined interventions, i.e., collagen with resistance exercise, vitamin K2, or bisphosphonates, to determine additive or synergistic effects.
4. Use economic evaluations to ascertain the cost-effectiveness of collagen peptide supplementation for osteoporosis prevention and fracture prevention in older age groups.
5. Develop response biomarkers to identify those most likely to benefit from collagen supplementation and so offer a precision nutrition approach.

## 6 Conclusion

This meta-analysis shows that collagen peptide supplementation, especially when combined with calcium and vitamin D, significantly enhances bone mineral density and could make a contribution to preventing fracture in populations at risk, such as postmenopausal women. The impacts are greatest at the spine and femoral neck, with moderate to large standardized mean differences in studies. Supplementation with collagen also shows consistent effects on bone turnover markers and indirectly can help in musculoskeletal well-being through improved muscle strength and mobility. Despite high heterogeneity in some fracture-related outcomes, results for bone metabolism and muscle outcomes were very consistent. Overall, collagen peptides are a fascinating addition to standard bone health therapy.
